# Performing meta-analysis with incomplete statistical information in clinical trials

**DOI:** 10.1186/1471-2288-8-56

**Published:** 2008-08-18

**Authors:** Jianbing Ma, Weiru Liu, Anthony Hunter, Weiya Zhang

**Affiliations:** 1 School of Electronics, Electrical Engineering and Computer Science, Queen's University Belfast, Belfast, BT7 1NN, UK; 2 Department of Computer Science, University College London, Gower Street, London, WC1E 6BT, UK; 3 Academic Rheumatology, Medical & Surgical Sciences, City Hospital, Nottingham, NG5 1PB, UK

## Abstract

**Background:**

Results from clinical trials are usually summarized in the form of sampling distributions. When full information (mean, SEM) about these distributions is given, performing meta-analysis is straightforward. However, when some of the sampling distributions only have mean values, a challenging issue is to decide how to use such distributions in meta-analysis. Currently, the most common approaches are either ignoring such trials or for each trial with a missing SEM, finding a similar trial and taking its SEM value as the missing SEM. Both approaches have drawbacks. As an alternative, this paper develops and tests two new methods, the first being the prognostic method and the second being the interval method, to estimate any missing SEMs from a set of sampling distributions with full information. A merging method is also proposed to handle clinical trials with partial information to simulate meta-analysis.

**Methods:**

Both of our methods use the assumption that the samples for which the sampling distributions will be merged are randomly selected from the same population. In the prognostic method, we predict the missing SEMs from the given SEMs. In the interval method, we define intervals that we believe will contain the missing SEMs and then we use these intervals in the merging process.

**Results:**

Two sets of clinical trials are used to verify our methods. One family of trials is on comparing different drugs for reduction of low density lipprotein cholesterol (LDL) for Type-2 diabetes, and the other is about the effectiveness of drugs for lowering intraocular pressure (IOP). Both methods are shown to be useful for approximating the conventional meta-analysis including trials with incomplete information. For example, the meta-analysis result of Latanoprost versus Timolol on IOP reduction for six months provided in [1] was 5.05 ± 1.15 (Mean ± SEM) with full information. If the last trial in this study is assumed to be with partial information, the traditional analysis method for dealing with incomplete information that ignores this trial would give 6.49 ± 1.36 while our prognostic method gives 5.02 ± 1.15, and our interval method provides two intervals as Mean ∈ [4.25, 5.63] and SEM ∈ [1.01, 1.24].

**Conclusion:**

Both the prognostic and the interval methods are useful alternatives for dealing with missing data in meta-analysis. We recommend clinicians to use the prognostic method to predict the missing SEMs in order to perform meta-analysis and the interval method for obtaining a more cautious result.

## Background

Clinical trials are widely used to test new drugs or to compare the effect of different drugs [2]. For example, many clinical trials have been carried out to investigate the efficacy of drugs for lowering intraocular pressure, such as travoprost, bimatoprost, timolol, and latanoprost, [3-11], and many to investigate the oral diabetes medication for adults with Type-2 diabetes [12-17]. Given that there is a huge number of trials available and reports on trials are very time consuming to read and understand, a systematic review of related trials is useful for medical practioners or other health care professionals to obtain an overall estimation about drugs/therapies of interest. Meta-analysis is the technique commonly used to summarize related trials results. Statistically, meta-analysis is in effect a process for merging sampling distributions (see its explanation in Preliminaries) into a single distribution.

When the full information of sampling distributions used in the trials is available, merging the results from these trials is usually a matter of systematic use of established techniques from statistics. However, in reality, some trial results reported in the literature are statistically incomplete. For instance, the standard error of mean (SEM) may be missing from a sampling distribution. A clinical trial with incomplete information is often abandoned, or, an SEM from a similar trial is used to substitute the missing data. Obviously, abandoning trials will decrease the power of a meta-analysis since trials that are eligible for analysis cannot be taken into account. On the other hand, if these trials are considered and a missing SEM is replaced by a substitute SEM, two major difficulties of this approach are inevitable. First finding a similar trial can be very difficult. Second there is no guarantee that there exists a similar trial with full information. Furthermore, measuring the similarity between trials is another issue that must be addressed. Therefore, an appropriate method is urgently needed to deal with sampling distributions with missing information when considering meta-analysis.

In this paper, we propose a prognostic method where missing SEMs are predicted from known information (SEMs) and an interval method where an interval is calculated in which the missing SEMs are assumed to be. These two methods are based on the assumption that the sampling distributions to be analyzed and merged are from the same population by random selection. In a sense, the two methods can be viewed as extremes. The prognostic method uses an estimate of the missing SEM, while the interval method aims at considering the best and worse cases. Of course, there are other reasonable intermediate procedures, for example, a Bayesian predictive approach is a very natural tool for dealing with missing information.

Significantly, our methods can also be used in the process of merging *between group differences *(almost all meta-analysis performs merging of between group differences). In fact, our methods can be applied to any results obtained from clinical trials, as long as these results (regarded as random variables) theoretically follow a sampling distribution.

## Methods

### Study examples

The study examples were drawn from two systematic reviews [1,17]. [17] reported a meta-analysis on drugs for patients with Type-2 diabetes and [1] reported a meta-analysis on lowering intraocular pressure for patients with open angle glaucoma or ocular hypertension.

In order to compare the results obtained from our methods with that from [17], we first attempted to collect all the original information on sampling distributions from each of the clinical trials papers used in [17]. After going through these papers, we found that we could not get the sampling distributions with full information from every single paper, as some of these papers did not provide the SEM values. We then applied our two methods to merge these (including partial) distributions and compared the merged result with that obtained in [17]. In contrast to [17], in [1] every sampling distribution used has full statistical information. To test the adequacy of our methods for dealing with missing information, we randomly selected a trial from [1] and deleted its SEM value. We then applied our two methods to recover this missing SEM value before merging this trial with the rest. Finally, we compared our results with that from [1].

### Preliminaries

In statistics, a normal distribution associated with a random variable is denoted as *X *~ *N *(*μ*, *σ*^2^). For the convenience of further calculations in the rest of the paper, we use notation *X *~ *N *(*μ*, *σ*) instead of *X *~ *N *(*μ*, *σ*^2^) for a normal distribution of variable *X*.

In statistics, random samples of individuals are often used as the representatives of the entire group of individuals (often denoted as a population) to estimate the values of some parameters of the population. The mean of variable *X *of the samples, when the sample size is reasonably large, follows a normal distribution. This distribution is typically referred to as a sampling distribution.

We use *X *~ *N *(*μ*, *SEM*) to denote a sampling distribution with mean value *μ *and standard error of mean *SEM*.

The basic merging rule for sampling distributions is as follows.

Let *X*_1 _~ *N *(*μ*_1_, *SEM*_1_) and *X*_2 _~ *N *(*μ*_1_, *SEM*_2_) be two sampling distributions, then the merged sampling distribution *X *~ *N *(*μ*, *SEM*) is given by

(1) $$\begin{array}{cc} \mu =\frac{\mu_{1}SEM_{2}^{2}+\mu_{2}SEM_{1}^{2}}{SEM_{1}^{2}+SEM_{2}^{2}}, & SEM=\sqrt{\frac{SEM_{1}^{2}SEM_{2}^{2}}{SEM_{1}^{2}+SEM_{2}^{2}}}. \end{array}$$

Equation 1 can be easily induced from *σ*_1 _= *σ*_2 _where *σ*_*i *_= *SEM*_*i*_$\sqrt{n_{i}}$ and *n*_*i *_is the number of samples of the *i*th trial (for *i *= 1, 2).

Conventionally, clinicians usually use notation $\omega_{i}=\frac{1}{SEM_{i}^{2}}$, thus Equation 1 can be rewritten as:

(2) $$\mu =\frac{\mu_{1}\omega_{1}+\mu_{2}\omega_{2}}{\omega_{1}+\omega_{2}},\omega =\omega_{1}+\omega_{2}.$$

It is easy to see that the merging rule is associative, thus this rule can be straightforwardly used to merge multiple sampling distributions. Let *X*_*i *_~ *N *(*μ*_*i*_, *SEM*_*i*_), 1 ≤ *i *≤ *k*, then for the merged sampling distribution *X *~ *N *(*μ*, *SEM*), we have

(3) $$\begin{array}{cc} \mu =\frac{\sum_{i=1}^{k}\mu_{i}\omega_{i}}{\sum_{i=1}^{k}\omega_{i}}, & \omega =\sum_{i=1}^{k}\omega_{i}. \end{array}$$

This is the classical merging method (i.e., meta-analysis) used by clinicians.

### The Prognostic Method

We propose a method to predict missing SEMs for sampling distributions from other distributions that have SEM values. Hereafter, we assume that there are *k *+ *l *trials altogether where *k *trials are with full information, i.e.,

(*μ*_1_, *SEM*_1_, *n*_1_),..., (*μ*_*k*_, *SEM*_*k*_, *n*_*k*_)

and *l *trials with partial information, i.e.,

(*μ*_*k*+1_, *n*_*k*+1_),..., (*μ*_*k*+*l*_, *n*_*k*+*l*_).

We want to get the merging result of those *k *+ *l *trials.

If we have both the SEM value and the sample size *n *from a trial, then we can obtain *σ *by equation *SEM *= $\frac{\sigma }{\sqrt{n}}$. Thus for *k *trials with

(*μ*_1_, *SEM*_1_, *n*_1_),..., (*μ*_*k*_, *SEM*_*k*_, *n*_*k*_)

we get *σ*_*i *_= *SEM*_*i*_$\sqrt{n_{i}}$, 1 ≤ *i *≤ *k*. Because it is assumed that these trials randomly select samples from the same population, these *σ*_*i*_s suggest what the real value *σ *could be. In fact, from the well-known *Error Theory*, we can use

$$\sigma^{*}=\frac{\sum_{i=1}^{k}\sigma_{i}}{k}$$

to replace the real *σ*. When *k *gets larger, σ* gets closer to the real *σ*. Therefore, for a sampling distribution without a standard error, we can use $SEM_{j}^{*}=\frac{\sigma^{*}}{\sqrt{n_{j}^{*}}}$ as its standard error where $n_{j}^{*}$ is the sample size of this sampling distribution.

To summarize, the prognostic method uses the following equation to predict the missing $SEM_{j}^{*}$ value for trial *j *with sample size $n_{j}^{*}$, given that for *k *trials, each of which has the *SEM*_*i *_value and the sample size *n*_*i*_.

$$SEM_{j}^{*}=\frac{\sum_{i=1}^{k}SEM_{i}\sqrt{n_{i}}}{k\sqrt{n_{j}^{*}}}.$$

Then we are able to use the standard meta-analysis method to merge trials with predicted SEM values and trials with full information.

### The Interval Method

In contrast to estimating a single value for a missing SEM as discussed above, in this section, we establish a method to estimate a reliable interval for a missing SEM. Then we propose the corresponding merging rule for this case.

For the *k *trials with

(*μ*_1_, *SEM*_1_, *n*_1_),..., (*μ*_*k*_, *SEM*_*k*_, *n*_*k*_),

we have *σ*_*i *_= *SEM*_*i *_$\sqrt{n_{i}}$, 1 ≤ *i *≤ *k*. Let

$$\begin{array}{cc} \sigma_{min}=min_{i=1}^{k}\sigma_{i} & \sigma_{max}=max_{i=1}^{k}\sigma_{i} \end{array}$$

It is intuitive to consider that the real *σ *value is in between *σ*_*min *_and *σ*_*max*_. Therefore for a sampling distribution without the standard error SEM*, we have $SEM^{*}\in \left[\frac{\sigma_{min}}{\sqrt{n^{*}}},\frac{\sigma_{max}}{\sqrt{n^{*}}}\right]$ where n* is the sample size of this trial.

It remains for us to investigate how to use this interval value for merging. For simplicity and illustration, first let us consider the case where there is only one trial with a missing SEM. Let *μ*^*k *^denote the weighted average of *μ*_1 _to *μ*_*k *_(the *k *trials with known SEMs), i.e.,

$$\mu^{k}=\frac{\sum_{i=1}^{k}\mu_{i}\omega_{i}}{\sum_{i=1}^{k}\omega_{i}}.$$

*μ*^*k *^is equivalent to the merged mean value for the *k *trials in meta-analysis. Let

$$\mu_{k+1}^{1}=\frac{\sum_{i=1}^{k}\mu_{i}\omega_{i}+n_{k+1}\mu_{k+1}/\sigma_{min}^{2}}{\sum_{i=1}^{k}\omega_{i}+n_{k+1}/\sigma_{min}^{2}}$$

and

$$\mu_{k+1}^{2}=\frac{\sum_{i=1}^{k}\mu_{i}\omega_{i}+n_{k+1}\mu_{k+1}/\sigma_{max}^{2}}{\sum_{i=1}^{k}\omega_{i}+n_{k+1}/\sigma_{max}^{2}},$$

then we have the following result.

Let *X*_*i *_~ *N *(*μ*_*i*_, *SEM*_*i*_), 1 ≤ *i *≤ *k*, denote the *i*th sampling distribution with sample size *n*_*i *_and *X*_*i*+1 _~ *N *(*μ*_*i*+1_, *SEM*_*i*+1_) denote the (*k *+ 1)th sampling distribution with sample size *n*_*i*+1 _where value *SEM*_*i*+1 _is unknown. Then the merged result *N *(*μ*, *SEM*) of the interval method is:

1. If *μ*_*k*+1_≤ *μ*^*k*^, then *μ *∈ [$\mu_{k+1}^{1}$, $\mu_{k+1}^{2}$]

2. If *μ*_*k*+1 _> *μ*^*k*^, then *μ *∈ [$\mu_{k+1}^{2}$, $\mu_{k+1}^{1}$]

and

$$SEM^{2}\in [\frac{1}{\sum_{i=1}^{k}\omega_{i}+n_{k+1}/\sigma_{min}^{2}},\frac{1}{\sum_{i=1}^{k}\omega_{i}+n_{k+1}/\sigma_{max}^{2}}]$$

The corresponding bounds of interval for *μ *depend on whether *μ*_*k*+1 _is larger or smaller than *μ*^*k*^. If *μ*_*k*+1 _is not bigger than *μ*^*k*^, then the lower (resp. upper) bound of its interval $\mu_{k+1}^{1}$ (resp. $\mu_{k+1}^{2}$) is obtained from the result of merging *k *+ 1 trials by the traditional meta-analysis method where the (*k *+ 1)th SEM is assumed to be $\frac{\sigma_{min}}{\sqrt{n_{k+1}}}\left(\text{resp}.\frac{\sigma_{max}}{\sqrt{n_{k+1}}}\right)$. On the other hand, if *μ*_*k*+1 _is larger than *μ*^*k*^, then the lower (resp. upper) bound is obtained from the result of merging *k *+ 1 trials by the traditional meta-analysis method where the (*k *+ 1)th SEM is assumed to be $\frac{\sigma_{max}}{\sqrt{n_{k+1}}}\left(\text{resp}.\frac{\sigma_{min}}{\sqrt{n_{k+1}}}\right)$

Similarly, for *l *trials with incomplete statistical information, we define

$$\mu_{k+l}^{1}=\frac{\sum_{i=1}^{k}\mu_{i}\omega_{i}+\sum_{i=k+1}^{k+l}n_{i}\mu_{i}/\sigma_{i}^{2}}{\sum_{i=1}^{k}\omega_{i}+\sum_{i=k+1}^{k+l}n_{i}/\sigma_{i}^{2}}$$

and

$$\mu_{k+l}^{2}=\frac{\sum_{i=1}^{k}\mu_{i}\omega_{i}+\sum_{i=k+1}^{k+l}n_{i}\mu_{i}/{\sigma^{\prime }}_{i}^{2}}{\sum_{i=1}^{k}\omega_{i}+\sum_{i=k+1}^{k+l}n_{i}/{\sigma^{\prime }}_{i}^{2}}.$$

∀*i*, *k *+ 1 ≤ *i *≤ *k *+ *l*, we let

$$\sigma_{i}=\sigma_{min},{\sigma^{\prime }}_{i}=\sigma_{max},\text{ if }\mu_{i}\leq \mu^{k},$$

and

$$\sigma_{i}=\sigma_{max},{\sigma^{\prime }}_{i}=\sigma_{min},\text{ if }\mu_{i}>\mu^{k},$$

then we have the following result.

Let *X*_*i *_~ *N *(*μ*_*i*_, *SEM*_*i*_), 1 ≤ *i *≤ *k *+ *l*, denote the *i*th sampling distribution with sample size *n*_*i *_such that *SEM*_*i *_is assumed missing when *i *> *k*, then the merged result *N *(*μ*, *SEM*) applying the interval method to these *k *+ *l *trials is:

$$\mu \in [\mu_{k+l}^{1},\mu_{k+l}^{2}],$$

and

$$SEM^{2}\in [\frac{1}{\sum_{i=1}^{k}\omega_{i}+\sum_{i=k+1}^{k+l}n_{i}/\sigma_{min}^{2}},\frac{1}{\sum_{i=1}^{k}\omega_{i}+\sum_{i=k+1}^{k+l}n_{i}/\sigma_{max}^{2}}].$$

### Merging of the Differences between Groups

In clinical trials, the *between group difference *of two drugs/therapies about two groups is frequently used. Suppose that the numbers of patients in the two groups are the same (majority of clinical trials allocate (approximately) the same number of patients in two contrast groups) and we denote this number as *n*. Assume that the first group gives a sampling distribution of the effect of one drug as *X*_1 _~ *N *(*μ*_1_, *SEM*_1_) and the second group about another drug as *X*_2 _~ *N *(*μ*_2_, *SEM*_2_). Conventionally, the between group difference (of the two drugs involved) is calculated as

$$X\sim N(\mu_{1}-\mu_{2},\sqrt{SEM_{1}^{2}+SEM_{2}^{2}}).$$

Thus we get

$$SEM=\sqrt{SEM_{1}^{2}+SEM_{2}^{2}}=\frac{\sqrt{\sigma_{1}^{2}+\sigma_{2}^{2}}}{\sqrt{n}}.$$

Let $\sigma =\sqrt{\sigma_{1}^{2}+\sigma_{2}^{2}}$ be a fixed value since *σ*_1 _and *σ*_2 _are fixed by the selected populations, we get *SEM *= $\frac{\sigma }{\sqrt{n}}$. Therefore, the two methods proposed in the previous sections are also suitable in the process of merging the between group differences.

## Results and discussion

### Case Study of Drugs for Type-2 Diabetes

In this section, we use the data from clinical trials on medication for adults with Type-2 diabetes as our first case study.

Many research papers and reports have been published to show the effectiveness of various oral medication for Type-2 diabetes ([12-16], etc.). For oral medication of Type-2 diabetes, the meta-analysis in [17] compared each pair of drugs on systolic blood pressure (SBP for short), diastolic blood pressure (DBP), low density lipoprotein cholesterol (LDL-C) and high density lipoprotein cholesterol (HDL-C), etc.

In this section, we consider the between group difference on the effectiveness of pairs of drugs for lowering LDL-C. In [17], there were 14 sets of results comparing between group difference of 14 pairs of drugs on lowering LDL-C. We selected four sets among these 14 sets for our case study, because each of them contains more than four trials so that our merging method can be applied more adequately. Our data were obtained from the original papers, most of which are the same as the data used in [17], however some data used in [17] are different from what we have found in the original papers. When this happens, we will state it clearly in the examples.

The drugs being compared in the selected four groups are

1. Thiazolidinedione versus Metformin (Example 1),

2. Triazolidinedione versus second generation Sulfonylureas (Example 2),

3. Metformin versus Metformin plus second generation Sulfonylureas (Example 3),

4. Second generation Sulfonylureas versus Metformin plus second generation Sulfonylureas (Example 4).

**Example 1 ***Drugs for reduction of low density lipoprotein (LDL for short) were studied by many papers. They compared the LDL-C reduction between different trial groups. For example, to compare drugs Thiazolidinedione and Metformin, we obtained the following (in mg/dL) sampling distributions from four trials*.

[18]: *X*_*Pa *_~ *N *(13.3, *SEM*_*Pa*_) *with n *= 102.

[12]: *X*_*LT *_~ *N *(7.8, 20.5) *with n *= 20.

[19]: *X*_*Han *_~ *N *(10.1, 2.7) *with n *= 320.

[20]: *X*_*Sch *_~ *N *(15.2, *SEM*_*Sch*_) *with n *= 597.

*Here n is the size of samples (number of patients) in each group of the trial, and SEM*_*Pa *_*and SEM*_*Sch *_*stand for the missing values (SEM values) from their respective trials data*.

*The meta-analysis results obtained by using prognostic method, interval method, and traditional method for incomplete trials information are provided in *Table 1.

**Table 1:** Thiazolidinedione vs. Metformin

Missing	P	Int	Inc
	(*X*_*P*_)	(*X*_*I*_)	(*X*_*Inc*_)
*X*_*Pa*_, *X*_*Sch*_	12.56 ± 1.91	[11.94, 13.39] ± [1.54, 2.14]	10.1 ± 2.68

*In *Table 1, *the first column shows the SEMs of X*_*Pa *_*and X*_*Sch *_*are missing. The second column shows that the result obtained by the prognostic (P for short) method is X*_*p *_~ *N *(12.56, 1.91) *(denoted as *12.56 ± 1.91 *in *Table 1*). The third column stands for the result obtained by the interval method (Int for short) and the last column stands for the result given by the traditional method on meta-analysis with incomplete trials information (Inc for short), i.e., removing trials with incomplete information. The rest of the tables in this case study follow the same notations and explanations*.

*Compared to X*_*BW *_~ *N *(12.5,1.89) [17]*which is the meta-analysis result when all information is complete, obviously, with two SEM values missing, X*_*P *_*is still very close to X*_*BW *_.* Therefore, X*_*P *_*can be used to replace X*_*BW *_* for this set of trials. In addition, we also have *12.5 ∈ [11.94, 13.39] *and *1.89 ∈ [1.54, 2.14] *which indicate that X*_*I *_*is a good estimation*.

**Example 2 ***To compare Thiazolidinedione and second generation Sulfonylureas, we obtained the following sampling distributions (in mg/dL) from five trials*.

[12]: *X*_*LT *_~ *N *(10.5, 14.44) *with n *= 20.

[13]: *X*_*CM *_~ *N *(11.31, 1.59) *with n *= 620.

[14]: *X*_*TJ *_~ *N *(6.63, *SEM*_*TJ*_) *with n *= 100.

[15]: *X*_*PM *_~ *N *(5, 6.04) *with n *= 86.

[16]: *X*_*MC *_~ *N *(14.6, *SEM*_*MC*_) *with n *= 315.

*There are two missing SEM values*.

*The meta-analysis results for this example are provided in *Table 2.

**Table 2:** Triazolidinedione vs. second generation Sulfonylureas

Missing	P	Int	Inc
	(*X*_*P*_)	(*X*_*I*_)	(*X*_*Inc*_)
*X*_*TJ*_, *X*_*MC*_	11.35 ± 1.32	[10.91, 11.88] ± [1.20, 1.38]	10.9 ± 1.53

*In this example, X*_*P *_*is reasonably close to X*_*BW *_*where X*_*BW *_~ *N *(10.4,1.61) [17]. *However X*_*Inc *_*is closer*.

**Example 3 ***To compare Metformin and Metformin plus second generation Sulfonylureas, we looked at another set containing six trials with sampling distributions as follows (in mg/dL)*.

[21]: *X*_*Ga *_~ *N *(-10.2, *SEM*_*Ga*_) *with n *= 168.

[22]: *X*_*Go *_~ *N *(-7.0, 5.18) *with n *= 60.

[23]: *X*_*Ma *_~ *N *(-3.9, 4.11) *with n *= 103.

[24]: *X*_*DeF *_~ *N *(2.0, 2.8) *with n *= 186.

[25]: *X*_*H*94 _~ *N *(-3.1, 3.6) *with n *= 30.

[26]: *X*_*H*91 _~ *N *(8.2, 5.8) *with n *= 15.

*There is only one missing SEM value. The meta-analysis result in *[17]* is X*_*BW *_~ *N *(-1.6, 2.53) *while we obtained the following results*.

*The meta-analysis results for this example are provided in *Table 3.

**Table 3:** Metformin vs. Metformin plus second generation Sulfonylureas

Missing	P	Int	Inc
	(*X*_*P*_)	(*X*_*I*_)	(*X*_*Inc*_)
*X*_*Ga*_	-3.82 ± 1.43	[-6.11, -2.88] ± [1.15, 1.53]	-0.73 ± 1.74

*Some data (sampling distributions of *[23,25,26]*) used in *[17]* are somehow different from the data we found from the original papers. Naturally, there is a margin between the merged sampling distribution from our methods and that from the meta-analysis. Therefore, it is difficult to compare these results*.

**Example 4 ***A set of seven trials was analyzed in *[17]* to compare the second generation Sulfonylureas versus Metformin plus second generation Sulfonylureas. We obtained the following seven sampling distributions (in mg/dL) from them*.

[21]: *X*_*Ga *_~ *N *(-2.2, *SEM*_*Ga*_) *with n *= 168.

[22]: *X*_*Go *_~ *N *(-0.2, 4.6) *with n *= 60.

[23]: *X*_*Ma *_~ *N *(3.9, 4.72) *with n *= 103.

[27]: *X*_*Gr *_~ *N *(10.14, 10.17) *with n *= 87.

[24]: *X*_*DeF *_~ *N *(11.0, 2.83) *with n *= 186.

[25]: *X*_*H*94 _~ *N *(7.41, 4.22) *with n *= 30.

[26]: *X*_*H*91 _~ *N *(19.89, 5.54) *with n *= 15.

*There is one SEM value missing. The results from our methods are given in *Table 4.

**Table 4:** Second generation Sulfonylureas vs. Metformin plus second generation Sulfonylureas

Missing	P	Int	Inc
	(*X*_*P*_)	(*X*_*I*_)	(*X*_*Inc*_)
*X*_*Ga*_	6.20 ± 1.57	[2.79, 7.96] ± [1.21, 1.73]	8.56 ± 1.78

*It appears that X*_*P *_*is not so close to X*_*BW *_*where X*_*BW *_~ *N *(8.1,2.55) [17]. *Once again, some data (sampling distributions of *[23,25,26]*) used in *[17]* are not the same as given in the original papers. So effectively, we had to use different data to perform the merging of these several trials*.

### Case Study of IOP Reduction

In the above subsection, we applied our methods to approximate the traditional meta-analysis when some SEM values are missing. Because some of the data we found in the original papers are different from that used in the meta-analysis paper [17], it is hard to judge our approaches in some cases. In order to further validate our approaches experimentally, in this section, we consider and apply our methods to a set of trials with full statistical information. In other words, every trial we consider will have both the mean and the SEM value in its sampling distribution. To validate how accurate our two approaches are for predicting an SEM value, we deleted an SEM value from a trial selected randomly from a set of trials, and applied our methods to predict it. We then applied the meta-analysis method to merge the trial with the predicted SEM value together with the rest of trials in the group to see how close this new result is to the original meta-analysis result. Furthermore, as the traditional method for trials with incomplete information always abandons trials with incomplete information, we also compared our methods with this traditional method.

We considered four sets of trials used in [1] on IOP reduction. [1] reported the meta-analysis of some trials about between group difference of IOP reduction from baselines using drugs Latanoprost and Timolol.

**Example 5 ***(one week results) Three papers on clinical trials about IOP reduction gave the following three sampling distributions comparing Latanoprost and Timolol for one-week trial duration*.

[28]: *X*_1 _~ *N *(8.5, 8.24) *with n *= 46.

[29]: *X*_2 _~ *N *(5.62, 7.91) *with n *= 15.

[30]: *X*_3 _~ *N *(6.85, 4.01) *with n *= 20.

*The traditional meta-analysis in *[1]* produced X*_1*w *_~ *N *(6.90, 3.28). *We deleted the SEM value from one of these trials in turn and applied our methods to predict it. Then we calculated the merged sampling distribution involving this predicted SEM value. The results are summarized in *Table 5.

**Table 5:** Latanoprost vs. Timolol: one week

Missing	P	Int	Inc
	$X_{P}^{i}$	$X_{I}^{i}$	$X_{Inc}^{i}$
*X*_1_	7.55 ± 2.53	[7.33, 7.83] ± [2.12, 2.80]	6.60 ± 3.57
*X*_2_	6.97 ± 3.37	[6.58, 7.07] ± [2.84, 3.49]	6.85 ± 4.01
*X*_3_	6.96 ± 4.91	[6.94, 6.97] ± [4.38, 5.19]	7.00 ± 5.71

*In *Table 5,* the second row starting with "X*_1_*" means that if the SEM value of X*_1 _*is missing, then from the prognostic method, we get *$X_{P}^{1}$ ~ *N *(7.55, 2.53),* from the interval method, we get *$X_{I}^{1}$ ~ *N *([7.33, 7.83], [2.12, 2.80]),* and from the traditional method for trials with incomplete information, we get *$X_{Inc}^{1}$ ~ *N *(6.60, 3.57).* Other rows are explained similarly. We can see that *$X_{P}^{i}$*s*, 1 ≤ *i *≤ 3,* are close to the result obtained by the traditional method*.

**Example 6 ***(one month results) Three papers on clinical trials about IOP reduction gave the following three sampling distributions comparing Latanoprost and Timolol for one month duration*.

[28]: *X*_1 _~ *N *(11.0, 6.05) *with n *= 46.

[31]: *X*_2 _~ *N *(5.2, 1.66) *with n *= 184.

[32]: *X*_3 _~ *N *(0.4, 2.17) *with n *= 294.

*The traditional meta-analysis gives X*_1*m *_~ *N *(3.77, 1.29).* The results are summarized in *Table 6. $X_{P}^{i}$*s*, 1 ≤ *i *≤ 3,* are also very close to the result obtained by the traditional method*.

**Table 6:** Latanoprost vs. Timolol: one month

Missing	P	Int	Inc
	$X_{P}^{i}$	$X_{I}^{i}$	$X_{Inc}^{i}$
*X*_1_	4.05 ± 1.26	[3.84, 4.46] ± [1.22, 1.28]	3.43 ± 1.32
*X*_2_	2.81 ± 1.67	[2.73, 2.89] ± [1.64, 1.69]	1.61 ± 2.04
*X*_3_	3.38 ± 1.21	[2.49, 4.00] ± [1.01, 1.33]	5.61 ± 1.60

**Example 7 ***(three months results) Five papers on clinical trials about IOP reduction gave the following five sampling distributions comparing Latanoprost and Timolol for three months duration*.

[33]: *X*_1 _~ *N *(4.0, 3.11) *with n *= 267.

[34]: *X*_2 _~ *N *(5.41, 5.56) *with n *= 60.

[35]: *X*_3 _~ *N *(3.2, 4.03) *with n *= 36.

[31]: *X*_4 _~ *N *(7.8, 3.98) *with n *= 184.

[32]: *X*_5 _~ *N *(1.9, 2.12) *with n *= 294.

*The traditional meta-analysis gives X*_3*m *_~ *N *(3.52, 1.44).* The results are summarized in *Table 7. *Once again*, $X_{P}^{i}$*s*, 1 ≤ *i *≤ 5,* are close to the result obtained by the traditional method*.

**Table 7:** Latanoprost vs. Timolol: three months

Missing	P	Int	Inc
	$X_{P}^{i}$	$X_{I}^{i}$	$X_{Inc}^{i}$
*X*_1_	3.58 ± 1.35	[3.51, 3.72] ± [1.09, 1.46]	3.39 ± 1.62
*X*_2_	3.53 ± 1.43	[3.47, 3.76] ± [1.34, 1.46]	3.38 ± 1.49
*X*_3_	3.55 ± 1.51	[3.54, 3.56] ± [1.49, 1.52]	3.57 ± 1.54
*X*_4_	3.99 ± 1.36	[3.59, 4.98] ± [1.17, 1.43]	2.88 ± 1.54
*X*_5_	3.77 ± 1.54	[2.93, 4.07] ± [1.14, 1.66]	4.91 ± 1.96

**Example 8 ***(six months results) Four papers on clinical trials about IOP reduction gave the following four sampling distributions comparing Latanoprost and Timolol for six months duration*.

[33]: *X*_1 _~ *N *(4.8, 3.14) *with n *= 267.

[36]: *X*_2 _~ *N *(7.1, 1.58) *with n *= 268.

[35]: *X*_3 _~ *N *(4.5, 5.23) *with n *= 36.

[32]: *X*_4 _~ *N *(1.5, 2.14) *with n *= 294.

*The traditional meta analysis gives X*_3*m *_~ *N *(5.05, 1.15).* The results are summarized in *Table 8. *Finally*, $X_{P}^{i}$*s*, 1 ≤ *i *≤ 4,* are also close to the result obtained by the traditional method*.

**Table 8:** Latanoprost vs. Timolol: six months

Missing	P	Int	Inc
	$X_{P}^{i}$	$X_{I}^{i}$	$X_{Inc}^{i}$
*X*_1_	5.00 ± 1.04	[4.98, 5.02] ± [0.97, 1.08]	5.09 ± 1.24
*X*_2_	4.15 ± 1.38	[3.72, 4.64] ± [1.26, 1.48]	2.75 ± 1.68
*X*_3_	5.06 ± 1.16	[5.04, 5.07] ± [1.14, 1.17]	5.08 ± 1.18
*X*_4_	5.02 ± 1.15	[4.25, 5.63] ± [1.01, 1.24]	6.49 ± 1.36

By comparing these results, we can conclude that the more trials we have in an example, the closer our result is to the traditional meta-analysis method. This is because when the number of trials included in a meta-analysis gets larger, the error of meta-analysis gets smaller. Therefore, the predicted SEM value from the prognostic method gets closer to the real SEM value and the intervals used in the interval method have a better chance to contain the real values.

## Discussion

Here we mainly analyze the results obtained from the IOP examples. Since we have stated that some data used in [17] are different from the data we found in the original papers, it would be difficult to compare those results objectively.

First, we found that the results obtained from the prognostic method are closer to the true results. It implies that the prognostic method is truly applicable. Although the interval method gives an interval instead of a single value, the interval provides a clear indication as to where the real value could be, and so it is an alternative to supplement the missing value.

Second, we found that the prognostic method is superior to the traditional method for trials with incomplete information which abandons trials with missing information. In fact, we can see that although in some cases, the results obtained by the traditional method for trials with incomplete information are also close to the true results, in some other cases, they deviate from the true results significantly, such as cases like in Table 6, when the SEM of *X*_2 _is missing, in Table 7, that of *X*_4 _or *X*_5 _is missing, and in Table 8, that of *X*_2 _or *X*_4 _is missing. That is, the prognostic method is more stable and precise than the traditional method for incomplete trial information. This result is not surprising because the prognostic method takes into account all the eligible trials. On the contrary, if the SEM value of a trial with a large/small mean value is missing, then the traditional method for trials with incomplete information will abandon this trial and naturally gets a result with smaller/higher mean value than the true one. However, the prognostic method does not abandon this trial, and instead it predicts a value for the missing one. Therefore, the final combined result is closer to the real result of meta-analysis. This is also illustrated by Examples 5,6,7,8, etc, except the case of the missing SEM of *X*_1 _in Example 5.

Dealing with missing data in statistics, especially in meta-analysis is a very important issue (e.g., [37-39]). However, there are hardly any papers focusing on missing standard errors. This paper thus provides some fresh results about how to deal with this situation. Our assumption that the populations should be the same or similar intuitively follows the well known Missing-at-random (MAR) assumption, except that MAR mainly focuses on repeatedly measured data while ours are focusing on different trials. With this assumption, the prognostic and the interval methods are easily induced. Generally speaking, the prognostic method is related to the regression imputation and the interval method is to some extent a concrete implementation of the best/worst case analysis [40] with our assumption and situations.

A small caveat of the prognostic and the interval methods is that when the input data are imprecise (as shown in Examples 3 and 4), we may obtain worse results than that from the traditional method for incomplete information. This is because when some input data are imprecise, the prognostic method will get wrong predictions for missing SEMs, hence obtain worse meta-analysis results whilst the traditional method for incomplete trial information simply abandons trials with incomplete information, avoiding any wrong predictions and therefore may obtain better results. In addition, in Example 5, when the SEM value of *X*_1 _is missing, we found that the result obtained by the prognostic method is not as accurate as that obtained by the traditional method for incomplete information. This is because the SEM value of *X*_1 _is exceptionally larger than the others, i.e., as the sample size of *X*_1 _is greater than those of *X*_2 _and *X*_3_, it is expected (according to our assumption of same population) that the SEM of *X*_1 _should be smaller, however, it is not. So the prognostic method performs less effectively here.

## Conclusion

Methods to deal with statistical information in clinical trials for which the SEMs values are missing is a neglected area in the literature. An obvious solution is to ignore a trial with missing SEM values. Our methods provide an application tool to solve this problem by effectively predicting missing SEMs or providing applicable intervals for the missing SEMs. The prognostic method can be used to obtain a result in a commonly accepted format, e.g., sampling distribution, whilst the interval method, although seemingly not so straightforward, provides a safer and also informative result.

## Competing interests

The authors declare that they have no competing interests.

## Authors' contributions

JM, WL and AH contributed to the analysis methods and the collection of materials on case studies. WZ contributed to the analysis methods and the evaluation of these new methods with respect to the traditional meta-analysis method and the traditional method for dealing with missing values. All authors read and approved the final manuscript.

## Pre-publication history

The pre-publication history for this paper can be accessed here:


